# Diode Green Laser in the Lid Trichiasis Treatment

**DOI:** 10.18502/jovr.v16i3.9428

**Published:** 2021-07-29

**Authors:** Raquel Galvão Bezerra, Roberta Lílian Fernandes de Sousa Meneghim, Carlos Roberto Padovani, Silvana Artioli Schellini

**Affiliations:** ^1^Department of Ophthalmology, São Paulo State University (UNESP), Faculty of Medicine of Botucatu, 18618687, Botucatu, São Paulo, Brazil; ^2^Department of Biostatistics, São Paulo State University (UNESP), Institute of Biosciences of Botucatu, 18618687, Botucatu, São Paulo, Brazil

**Keywords:** Ablation Techniques, Diode Lasers, Success Rate, Trichiasis

## Abstract

**Purpose:**

To validate the standard values and evaluate the success rate in the treatment of minor and major trichiasis using thermoablation with a diode green laser.

**Methods:**

In this interventional prospective study, individuals with minor or major trichiasis who were treated with thermoablation using diode green laser were included. The patients' mean age was 72.1 years; the majority were females (54.1%) and Caucasian (98%). The parameters of the diode laser were wavelength of 532 nm, application time of 200 ms, target size of 50 μm, interval between the shots 150 to 200 ms, and power of 600 to 750 mW. The number of shots was defined by the depth of ablation sufficient to reach the pilus hair bulb. The patients were evaluated by slit-lamp every 3 to 4 months, for up to 15 months. The treatment success rate and the association between variables were analyzed.

**Results:**

The study sample was comprised of 98 patients with 135 affected lids and 337 lashes with trichiasis. Minor trichiasis (91.8%), unilateral trichiasis (67.3%), trichiasis affecting the lower eyelid (85.9%), and trichiasis resulting from blepharitis (64.3%) were the most common presentation profiles. The overall cure rate at the end of the study was 85%, with 69% being cured with a single session and 82.8% with two treatment sessions.

**Conclusion:**

Thermoablation using a diode green laser applying the specified parameters to treat minor and major trichiasis is effective and results in high cure rate.

##  INTRODUCTION

Laser thermoablation is a simple procedure, capable of permanently treating overlapping trichiatic lashes, with minimal damage to the lid tissues and with a low risk of complications.^[1,2,3]^ Laser thermoablation has already proved to be a good modality of treatment for trichiasis.^[1,2,3]^ Argon laser, ruby laser, and diode laser are all suggested for the treatment of trichiasis;^[4,5,6]^ however, the majority of studies have reported on argon laser treatment for trichiasis.^[1,4,7,8,9,10,11,12,13,14,15,16]^

Although the diode laser is less popular, three previous studies report good results.^[6,17,18]^ However, standard laser treatment parameters have not been established and success rates vary, which impedes popularity of the procedure. This study aimed to validate the standard values and success rate with the application of diode green laser for the treatment of minor and major trichiasis in order to reinforce the good results with this technique.

##  METHODS

The Ethics Research Committee of the Faculty of Medicine of Botucatu (FMB), São Paulo, Brazil approved this study. All patients signed a consent form prior to treatment. This longitudinal, prospective, interventional study enrolled patients from July 2016 to April 2017 with minor (less than five lashes touching the ocular surface) or major (five or more lashes touching the ocular surface) trichiasis, who underwent thermoablation of the lashes using a diode green laser at Clinical Hospital of FMB, Brazil.

Individuals were excluded from the study if they had more than 10 trichiatic lashes, had distichiasis, an inability to position themselves in the slit-lamp to receive treatment, less than six months follow-up, and those who refused to participate in the study.

Prior to laser delivery, a combination of proxymetacaine hydrochloride 5mg/ml (AnestalconⓇ, Novartis Biociências, São Paulo, SP) and lidocaine ointment 25mg/g + prilocaine 25mg/g (MedicaínaⓇ 5%, Cristália, Itapira, SP) were applied to the region of treatment. The patient was positioned to receive the laser through a slit-lamp adapted to a diode laser with green light emission (Visulas 532s Green Laser System; Zeiss, Jena, Germany), with fixation of the chin and forehead, eye aligned to the center of the equipment, palpebral margin everted with cotton tip to better expose the lashes to be treated and oriented to look in the direction opposite to the region planned for laser application.

The diode green laser parameters were standardized and defined from a previous study of the argon laser,^[16]^ with modifications based on empirical observations as follows: application time of 200 ms, target of 50 μm, range of 150 to 200 ms, power of 600 to 750 mW.

The laser light beam was applied by around 10 different doctors who received training in the procedures before starting the study. Lashes were treated one by one and no more than 10 lashes were destroyed in each session. The laser beam was focused parallel to the base of the abnormal hair follicle to be treated. The initial application vaporized the lash and applications were done until a crater was created in the lid margin. The number of shots was defined by the depth of the crater sufficient to reach the hair bulb, considering ablation of 2.5 mm for lashes in the upper eyelid and 1.5 mm for those located in the lower lid. The depth of 2.5 mm was considered based on the bevel length of an insulin needle.^[16]^ After application, the treated area received neomycin sulfate, polymyxin B sulfate, and dexamethasone ointment (MaxitrolⓇ, Alcon, São Paulo, SP) twice daily for seven days.

The patients were re-evaluated by a slit-lamp three to four months after the laser session. In case of relapse or recurrence, new applications were performed using the same parameters, with reassessments following the same periodicity until the end of the study.

The established criterion for successful treatment was the absence of trichiatic cilia in at least two consecutive evaluations. The success rate of the treatment was calculated from the number of laser sessions the patient received to obtain the cure. The association of success to other variables was analyzed.

Ophthalmologic and lid examination data were collected in an Excel spreadsheet (Microsoft Corp., Redmond, WA, USA). Data were collected on the location of the misdirected lashes, etiology of trichiasis and number of treated lashes, mean number of shots needed for ablation of the trichiatic lashes, and the outcomes (recurrence/relapse, or cure).

Data were analyzed with the Goodman homogeneity test, involving contrasts between multinomial populations, considering *P* < 0.05 and the Mann–Whitney test and Kruskal Wallis test complemented with Dunn's multiple comparisons for non-parametric variables, given the possibility of nonadherence of the variable to the normal distribution of probabilities.

##  RESULTS

The study comprised of 98 patients (130 eyes, 135 affected lids and 337 lashes with trichiasis). Sixty-six (67.3%) patients had unilateral involvement, the right side was affected in seventy-one (54.6%), and the lower lid was affected in one-hundred-sixteen (85.9%) patients. The treatment was applied to treat minor trichiasis in 124 (91.8%) lids and major trichiasis in 11 (8.2%) lids.

The mean age of the individuals was 72.1 ± 12.3 years; the majority were female (53/54.1%) and Caucasian (96/98%). The most commonly associated etiology was blepharitis (63/64.3%), idiopathic trichiasis (15/15.3%) followed by the association of blepharitis with ectropion (9/9.2%) or other causes [Table 1].

**Table 1:** Etiology of trichiasis in patients treated with diode green laser


**Etiology**	**Total**
Blepharitis	63 (64.3%)
Idiopathic	15 (15.3%)
Blepharitis + Ectropion	9 (9.2%)
Ectropion	4 (4.1%)
Trachoma	4 (4.1%)
Blepharitis + Entropion ${}^{*}$	2 (2.0%)
Entropion	1 (1.0%)
${}^{*}$ Some of these entropion may have elapsed from cicatricial trachoma				

There was no statistical significance comparing the variables age, sex, race, etiology, or systemic diagnosis with the presence or quantity of the number of lashes with trichiatic cilia (*P* > 0.05).

The average diode green laser shots needed to crater reach the depth of 2.5 mm in the upper eyelid or 1.5 mm in the lower was 60.7/cilia. The number of shots was statistically proportional to the number of misdirected lashes (*P* < 0.001), however, there was no significance compared to the number of shots with the sessions. Follow-up time ranged from 6 to 15 months, with participants having a minimum of one and a maximum of four sessions.

Eighty-seven patients completed follow-up and eleven (11.2%) patients did not. Hence, 87 patients comprised the study sample available for analysis. Success was observed in 74 (85%), with 60 (69%) cases achieving success in a single session, 12 (13.8%) with two sessions, 1 (1.1%) with three or four sessions. Treatment was unsuccessful in 13 (15%) patients who had relapses or recurrence at the last follow-up [Table 2]. There was a reduction from 3.0 to 0.9 trichiatic lashes per patient and from 2.5 to 0.7 eyelid lashes (*P* < 0.001) with only one treatment session.

There was no statistical significance (*P* > 0.05) for the comparison of successful treatment to the number of misdirected lashes. According to the etiology of trichiasis, the cure rate in the idiopathic etiology was higher (91.7%) than the others, while chronic inflammation of the lid margin was the cause with a lower cure rate (80.7%) (*P* < 0.05).

##  DISCUSSION

Although laser thermoablation is considered the effective treatment for trichiasis, there is still no standardized application pattern. Hence, we performed this study using the diode green laser (532 nm), time of application of 200 ms, target of 50 μm, interval of 150 to 200 ms, power of 600 to 750 mW, resulting in an 85% success rate. Elderly individuals with a mean age of 72.1 years, females, and Caucasian comprised the majority of our sample, similar to previous studies.^[5,13]^ Although males and fair skin were the most affected, those parameters were not relevant to success.^[6]^

The vast majority of our cases had minor trichiasis like other studies,^[8,10,13]^ with unilateral involvement in 67.3% of the patients and 85.9% of our patients had the lower eyelid affected, presumably because of greater exposure of the lower lid.^[4,5,6][10][16]^

Chronic blepharitis, scarring of the palpebral margin, and idiopathic etiology had the greatest association to trichiasis. Although trachoma is considered the main cause of trichiasis^[4,17]^ and bilateral and the upper eyelid pathology occur in patients with trichiasis associated to trachoma,^[6,19]^ it was detected in only 4.1% of our study group.

We included 98 patients treated with diode green laser with a follow-up period ranging from 6 to 18 months. These patients required between one to four treatment sessions for a successful outcome. However, 11.2% of the patients were non-compliant with the follow-up schedule. The majority of the sample was composed of elderly patients with limiting comorbidities, which may be a barrier to the adherence. Additionally, reduced patient compliance can occur due to pain or discomfort during application of the laser, despite instillation of topical anesthesia. Minimal or absent discomfort has been reported without anesthesia,^[10]^ with topical anesthesia,^[9,16,20]^ with injectable anesthesia,^[13,15]^ or with a combination of both.^[4,8]^ If patients are given the choice, they would prefer the injectable anesthesia.^[4]^

Different types of laser equipment and parameters can result in a variable rate of success for the treatment of trichiasis. Additionally, according to the manufacturer, the devices require calibration of the energy measurement at least yearly or earlier for optimal performance.^[21]^

Different parameters may lead to decreased laser light absorption by the melanin of the lash,^[22]^ which may explain diverse results. Only three other studies have been performed using the diode laser for treatment of trichiasis. One study used the same wavelength laser as the present study (532 nm) but with a higher power (up to 1,000 mW) and reported a lower success rate than ours (67.9% with two sessions).^[17]^

The diode green laser was used in previous studies with a longer wavelength of 806^[18]^ or 810 nm.^[16]^ However, the sample from one of the studies was small (22 patients) and thermoablation was used for patients with trachomatous trichiasis, with lower success rates,^[17]^ probably due to the chronic character of lid inflammation.^[15]^ Additionally, much lower energy of 20 J/cm² was used^[18]^ compared to our study since we used energies ranging from 600 to 750 mW, equivalent to 48 to 60 J/cm².

Treatment success was achieved in 69% of our cases with only one laser green diode application and the rate increased to 82.8% with two laser sessions, concurring with previous literature.^[6]^ With subsequent sessions, the success rate did not increase significantly.

The success rates using argon laser to treat trichiasis was similar to ours, reaching 80% with three sessions using 2,000 mW and 0.05 to 0.1 exposure time;^[9]^ or 85.2% with three sessions using 500mW and exposure time of 0.3 s;^[8]^ or 62.6% success with just one session of 1,000 or 1,200 mW and a target of 100 um.^[15]^ Success rates can be improved using four or five argon laser sessions, increasing success rates from 67.9% to 100%^[10]^ or 37.7% to 98.7%.^[16]^

Using ruby laser, complete success in trichiasis treatment occurred in 60% of cases,^[5]^ a much lower rate compared to 85% that we obtained with the diode green laser. However, few patients were included and only three shots per lash was applied,^[5]^ with a possibility to reduce success because the laser hardly reached the hair bulb.

The number of shots applied during the sessions in our study was directly proportional to the number of misdirected lashes, having more shots a direct association with the number of lashes altered per lid.^[15]^

Our results point to the good outcomes with diode green laser application enhanced by a reduction of number of trichiatic lashes/patient (3.0 to 0.9 trichiatic lashes per patient) and number of misdirected lashes per session (2.5 to 0.7 lashes/lid with only one session) (*P* < 0.001). A study of 44 patients reported slightly better results using higher energy density (between 57 and 70 J/cm²), observing reduction from 3.58 to 0.73 cilia/lid, equivalent to a 78.6% reduction with one session^[6]^ but the follow-up period was just three months.

We observed the highest success rate in idiopathic trichiasis (91.7%), similar to previous reports.^[8,15]^ Despite a few trachomatous trichiasis cases, the success rates may be significantly lower due to the fact that these individuals may present anatomical changes in the eyelid, making it difficult to reach the hair bulb,^[15]^ in addition to the chronic and persistent inflammation.

The present study had limitations including nonadherence of 11.2% of the participants and the fact that several ophthalmologists had applied the treatment, even though they were all trained and standardized prior to the study. The criteria for successful treatment and the success rate in our study were based on the relapse/recurrence of the trichiasis during follow-up. Although the definitions of recurrence and relapse differ, it was not possible to differentiate them because there was no photographic documentation in this study.

The strengths of our study were the prospective nature of the study, the high number of participants, the application of standardized parameters, the long follow-up period and the statistical analysis of the data that allowed a higher chance of success with the application of diode green laser for the treatment of trichiasis. The major advantages of diode green laser therapy are precision and selectivity of application. Diode laser application is an easy procedure, no need for infiltrative anesthesia resulting in mild inflammation and no scarring.

In conclusion, thermoablation using the diode green laser with a wavelength of 532 nm, application time of 200 ms, target of 50 μm, interval of 150 to 200 ms, power of 600 to 750 mW was effective for treating minor and major trichiasis, with success achieved in 85% of the patients. It took up to two sessions for successful treatment in most patients, with an insignificant increase in success with more sessions.

##  Financial Support and Sponsorship

Nil.

##  Conflicts of Interest

The authors do not have any conflicts of interest.
